# An improved expression and purification protocol enables the structural characterization of Mnt1, an antifungal target from *Candida albicans*

**DOI:** 10.1186/s40694-024-00174-5

**Published:** 2024-05-07

**Authors:** Patrícia Alves Silva, Amanda Araújo Souza, Gideane Mendes de Oliveira, Marcelo Henrique Soller Ramada, Nahúm Valente Hernández, Héctor Manuel Mora-Montes, Renata Vieira Bueno, Diogo Martins-de-Sa, Sonia Maria de Freitas, Maria Sueli Soares Felipe, João Alexandre Ribeiro Gonçalves Barbosa

**Affiliations:** 1 Laboratório de Biofísica Molecular, Departamento de Biologia Celular, Instituto de Ciências Biológicas, Universidade de Brasília, Brasília, 70910-900 Brazil; 2 Programa de Pós-graduação em Ciências Genômicas e Biotecnologia, Universidade Católica de Brasília, Brasília, 70790-160 Brazil; 3 Departmento de Biologia, División de Ciencias Naturales y Exactas, Universidad de Guanajuato, Guanajuato, 36050 Mexico; 4 Genesilico Biotech, Brasília, DF Brazil

**Keywords:** α-1,2-mannosyltransferase, *Candida albicans*, Glycosylation, Mnt1/Kre2, Structural properties, Drug target, Extracellular matrix, Biofilm, Drug resistance

## Abstract

**Background:**

*Candida albicans* is one of the most prevalent fungi causing infections in the world. Mnt1 is a mannosyltransferase that participates in both the cell wall biogenesis and biofilm growth of *C. albicans.* While the cell wall performs crucial functions in pathogenesis, biofilm growth is correlated with sequestration of drugs by the extracellular matrix. Therefore, antifungals targeting CaMnt1 can compromise fungal development and potentially also render *Candida* susceptible to drug therapy. Despite its importance, CaMnt1 has not yet been purified to high standards and its biophysical properties are lacking.

**Results:**

We describe a new protocol to obtain high yield of recombinant CaMnt1 in *Komagataella phaffii* using methanol induction. The purified protein’s identity was confirmed by MALDI-TOF/TOF mass spectroscopy. The Far-UV circular dichroism (CD) spectra demonstrate that the secondary structure of CaMnt1 is compatible with a protein formed by α-helices and β-sheets at pH 7.0. The fluorescence spectroscopy results show that the tertiary structure of CaMnt1 is pH-dependent, with a greater intensity of fluorescence emission at pH 7.0. Using our molecular modeling protocol, we depict for the first time the ternary complex of CaMnt1 bound to its two substrates, which has enabled the identification of residues involved in substrate specificity and catalytic reaction. Our results corroborate the hypothesis that Tyr209 stabilizes the formation of an oxocarbenium ion-like intermediate during nucleophilic attack of the acceptor sugar, opposing the double displacement mechanism proposed by other reports.

**Conclusions:**

The methodology presented here can substantially improve the yield of recombinant CaMnt1 expressed in flask-grown yeasts. In addition, the structural characterization of the fungal mannosyltransferase presents novelties that can be exploited for new antifungal drug’s development.

**Supplementary Information:**

The online version contains supplementary material available at 10.1186/s40694-024-00174-5.

## Background

Invasive fungal infections (IFIs) pose a growing global threat, afflicting over 1 million individuals annually [1, 2]. This reality is particularly acute for immunocompromised patients, including those undergoing organ transplants or receiving intensive care [3–7]. To make things worse, the use of broad-spectrum antibiotics further promotes susceptibility to IFIs [3, 8, 9]. Among the fungal pathogens, *Candida* ssp. alone account for 30–70% of IFI-related fatalities [10–12], with associated healthcare costs reaching billions of dollars [13, 14]. Recent reports have also highlighted the emergence of *Candida* infections in COVID-19 patients, extending their hospital stays and adding another layer to the public health dilemma [15].

*Candida* species commonly form biofilms that render them resistant to antimicrobial therapy [16]. The clinical impact of biofilm resistance is most severe for both vascular and surgically implanted devices infected with *Candida*, which can double patient mortality if not removed [17]. Unfortunately, the biofilm-active concentrations of current antifungals far exceed safe or achievable dosing regimens, thereby requiring the development of novel therapeutical approaches. The dimorphic fungus *Candida albicans* is the most prevalent pathogen in candidemia [18], boasting a remarkable adaptability that allows it to transition between harmless commensal and opportunistic pathogen. Due to it inhabiting various mucosal surfaces in the host, *C. albicans* can cause superficial and systemic infections, as well as chronic infections [13, 19, 20]. Nevertheless, non-*albicans Candida* species like *C. glabrata* and *C. parapsilosis* have increasingly challenged existing therapeutic options [13, 21].

Understanding the virulence mechanisms behind *Candida* infections is crucial for developing effective antifungal strategies. In this regard, the cell wall emerges as a critical battleground, for its structure is rich in carbohydrates and glycoproteins that perform pivotal functions in pathogenesis [22]. The outermost layer is formed primarily of mannoproteins carrying highly *O*- and *N*-linked glycans, known as mannans [23, 24]. Mannans are complex polymers consisting of D-mannose monomers connected by glycosidic bonds. Owing to the outermost position of mannans in the fungi wall, the mannosylation of cell wall-associated proteins dictates both fungal interaction with the host and resistance to immune defense mechanisms.

It has been shown that the main mechanism underlying *Candida* biofilm resistance to antifungals involves the sequestration of drugs by the extracellular matrix [25]. Investigations into the composition of the matrix have culminated in the recent identification of a novel mannan-glucan material consisting of α-1,2-branched α-1,6-mannan, β-1,6-glucan, and β-1,3-glucan [26]. Importantly, drug sequestration has been directly linked to the presence and abundance of this mannan–glucan complex in the matrix [27]. Small extracellular vesicles (exosomes) released by *Candida* during biofilm growth have been shown to contain carbohydrates identical to those found in the matrix, suggesting a direct link between vesicle cargo and matrix synthesis [16]. Notably, a significant portion of the proteins identified within these vesicles are also core components of the extracellular matrix, further solidifying this connection [27, 28].

The importance of α-1,2-mannan for cell wall synthesis has been well characterized [29, 30], but its potential role in drug resistance is a novel finding. The assembly of mannan polymers relies on mannosyltransferases, a specific set of multifunctional glycosyltransferases (GTs) that catalyze the glycosidic bond between mannose and acceptor residues [31, 32]. The reaction is made possible due to the phosphorylated donor sugar, which releases the phosphate group by the end of the glycosylation process [33]. Indeed, the proteome cargo of biofilm vesicles from *C. albicans* were shown to contain the mannosyltransferase Mnt1 [34], which is responsible for the synthesis of α-1,2-mannan in the cell wall [35, 36]. In this case, CaMnt1 was shown to be directly associated with the adhesion and dispersion mechanisms of *Candida* biofilm [34]. Orthologs of CaMnt1 have also been identified in the biofilm vesicles of *Candida tropicalis* and *Candida parapsilosis* (Interpro accessions C5M9H1 and G8BGU3, respectively), suggesting it has a conserved role in biofilm growth and community coordination [28]. Consequently, antifungals targeting CaMnt1 could both compromise fungal development [37, 38] and potentially render *Candida* biofilm susceptible to drug therapy [34].

GTs like CaMnt1 are classified according to their fold (GT-A or GT-B) and the stereochemical output of their catalysis, thereby grouping them into inverters or retainers depending on whether the newly formed glycosidic linkage retains the same anomeric configuration of the donor sugar or inverts it [33]. CaMnt1 has been annotated under the Kre2/Mnt1 family, also known as glycosyltransferase family 15 in the CAZy database [33, 39]. A total of nine proteins from this family have been identified in *S. cerevisiae* (ScKre2/Mnt1, ScKtr1, ScKtr2, ScKtr3, ScKtr4, ScKtr5, ScKtr6, ScKtr7, and ScYur1), and five in *Candida albicans* (CaMnt1, CaMnt2, CaMnt3, CaMnt4, and CaMnt5) [40, 41]. Orthologs of CaMnt1 and ScKre2/Mnt1 have also been identified in *Cryptococcus* spp, *Paracoccidioides* spp, and *Histoplama* spp. Given that CaMnt1 orthologs are present in fungi of medical importance but absent in humans [42], they represent an attractive target for new antifungals.

In this work, we describe protocol modifications that result in high yield of recombinant CaMnt1 expressed in flask-grown *Komagataella phaffii*, often still referred to by its obsolete name *Pichia pastoris*. The elevated degree of purity of CaMnt1 obtained using this purification protocol has enabled us to perform fluorescence and spectroscopy studies on the protein. In addition, we describe for the first time the ternary complex of CaMnt1 bound to its two substrates. Our findings may contribute to the development of new antifungal drugs targeting Mnt1 from *C. albicans*.

## Methods

### Heterologous expression of CaMnt1 in *Komagataella phaffii*

The producer clone was donated by Dr. Hector M. Mora Montes of University of Guanajuato, Mexico. The construction and selection were described by Díaz-Jiménez [32]. The *MNT1* gene (Genbank accession no. X99619) was optimized with *Komagataella phaffii* preferred codons and synthesized to obtain the recombinant protein containing only the soluble portion, corresponding to base pairs 91 to 1296. Then, the gene was subcloned into the pPICZαC vector, which was used to transform *K. phaffi*. A fresh colony of the transformant was inoculated into a 1 L flask containing 100 mL of BMGY medium [1% (w/v) yeast extract, 100 mM potassium phosphate buffer pH 6.0, 1.34% (w/v) YNB without amino acids, 4 × 10^− 5^% (w/v) biotin, and 1% (v/v) glycerol]. The culture was incubated at 250 rpm and 28 °C for 24 h. After this time, cells were harvested by centrifugation at 1500 x *g* for 5 minutes, and the culture medium was changed to BMMY [1% (w/v) yeast extract, 100 mM potassium phosphate buffer, 1.34% (w/v) YNB without amino acids, 4 × 10^− 5^% (w/v) biotin, and 1% (v/v) methanol], adjusting the volume until OD_600nm_ reached 5.0. Methanol was added to the culture twice a day, corresponding to 0.5% and 1%, in the morning and evening, respectively. Heterologous expression was maintained for 48 h at 250 rpm and 28 °C. Finally, the soluble fraction of recombinant protein was analyzed on SDS-PAGE 12% stained with Coomassie blue R-250 (Invitrogen, 2009) – with modifications.

### Protein purification

The expression system in *K. phaffii* was prepared in 1 L flasks containing 100 mL of culture. After 48 h of induction, the crude extract was centrifuged at 8000 x *g* for 20 min at 4 °C. The supernatant containing the recombinant protein was recovered and treated for pigment removal and volume reduction. First, 500 mL of the supernatant was concentrated 10-fold in Amicon® Ultra-30 kDa centrifugal filter, using a pressure system with nitrogen gas. The concentrated material was then dialyzed in 900 mL of 50 mM Tris HCl pH 8.0 buffer, then concentrated to 50 mL. After dialysis, the clarified supernatant was concentrated 16 times by ultracentrifugation in Amicon® Ultra-30 kDa, resulting in 3 mL. The protein purification was carried out by chromatographic methods using ÄKTA Pure system from G.E. Healthcare. In the first round, a size exclusion chromatography (SEC) was performed using a Superdex™ G-75 column. For SEC, 0.5 mL of sample was purified at a flow rate of 0.5 mL/min of buffer A (50 mM Tris HCl; 0.15 M NaCl; pH 8.0). The eluate fraction containing Mnt1 protein was then submitted to ion-exchange chromatography (IEX) using a HiTrap™ Q XL 1 mL column from G.E. Healthcare. The IEX purification step was performed using a gradient from 0.15 to 0.5 M of sodium chloride, with a flow rate of 0.5 mL/min. The fractions corresponding to the protein of interest were applied on the HiTrap™ desalting column for the removal of excess salt. All purification steps and resulting fractions were analyzed by 12% SDS-PAGE stained with Coomassie Brilliant Blue R-250.

### MALDI-TOF/TOF MS analysis

To confirm recombinant protein production and molecular weight estimation, MALDI-TOF/TOF (matrix-assisted laser desorption/ionization-time of flight) spectrometry analyses were performed on CaMnt1 after the purification steps. First, the highest purity band was extracted from the polyacrylamide gel and digested with trypsin Gold-Mass V582A (Promega) according to [43]. The resulting peptides were analyzed by AutoFlex Speed II MALDI-TOF/TOF mass spectrometer controlled by FlexControl 3.0 Software (Bruker Daltonics). Tryptic peptides were mixed (3:1, v/v) with α-cyano-4-hydroxycinnamic acid matrix solution (10 mg/mL, 50% CAN, 0.3% TFA) directly applied onto an MTP Anchor Chip 400/384 target plate (Bruker Daltonics) and dried at room temperature. Peptides monoisotopic masses were obtained in positive reflector mode ranging from 700 to 3500 m/z with external calibration using the Protein Calibration Standard II (Bruker Daltonics). Peptide MS/MS spectra were obtained by means of LIFT fragmentation. The software Flex Analysis3.0 (Bruker Daltoncs) and PepSeq (Waters) were used for mass spectrometric data analysis. Peptide primary structures were inferred by employing manual interpretation of fragmentation. The obtained sequences were then searched against the NCBI_nr_ protein database (www.ncbi.nlm.nih.gov) using the algorithm Blastp. The identified protein sequences were analyzed using the Peptide Mass tool from the ProtParam Server (www.expasy.org) to predict theoretical molecular weight.

### Circular dichroism (CD) assays

The circular dichroism measurements of CaMnt1 (0.11 mg/mL in 5 mM sodium citrate buffer [pH 5.0], 5 mM Tris HCl buffer [pH 7.0], and 5 mM glycine buffer [pH 9.5]) were carried out using the Jasco J-815 CD Spectropolarimeter (Jasco Corporation, Tokyo, Japan) equipped with a Peltier temperature controller (Jasco Analytical Instruments, Japan). Far-UV CD spectra from 200 to 260 nm were recorded using 0.1 cm quartz cuvettes with a data acquisition interval of 0.2 nm at 25 °C. Five successive scans were acquired, and their mean spectrum was recorded. The buffer spectrum was subtracted from each protein spectrum. The mean ellipticities were converted to molar ellipticity [θ] (deg.cm^2^/dmol) based on a mean molecular mass per residue of 115 Da [44]. The secondary structure content was determined using the CD Spectral deconvolution version 2.1 CDNN program [45].

The thermal stability assay of CaMnt1 was conducted at pH 5.0, 7.0 and 9.5. The ellipticity changes were monitored at λ_208_ nm in the temperature range of 25 to 95 °C, with the scan rate set at 0.2 °C/min. Concurrently, Far-UV CD spectra were recorded in the range of 200–260 nm, at 10 °C intervals with a data pitch of 0.2 nm. The unfolding curves were obtained by plotting the values of molar ellipticity versus temperature. The melting temperature (T_*m*_) was calculated from the nonlinear fitted unfolding curves using the Origin 8.1 software (Origin Lab Corporation, USA).

### Fluorescence spectroscopy

The fluorescence measurements of CaMnt1 were performed using the Jasco Spectrofluorimeter FP-650 (Jasco Corporation, Tokyo, Japan) coupled to a Peltier-type temperature controller (Jasco Analytical Instruments, Japan). The fluorescence spectra were recorded in the range of 300–400 nm at different pH’s, after excitation of tryptophan residues at 295 nm and 25 °C. The excitation and emission slits were adjusted to 5 nm, respectively [46].

The pH-dependent conformational changes of Mnt1 (0.036 mg / mL) were analyzed using 10 mM sodium citrate buffer (pH 4.0 ‒ 5.5), 20 mM Tris HCl buffer (pH 6.0 ‒ 9.0), and glycine buffer (pH 9.5) with a range difference of 0.5.

### Modeling and structural analyses

The search for homologous sequences to CaMnt1 was performed using the blastp algorithm (NCBI) against protein sequences deposited in the PDB database [47]. The structure of *S. cerevisiae* Kre2p/Mnt1p complexed with Mn^2+^ (MN), GDP and methyl-α-mannose (PDB 1S4P) was selected as a template [35]. Modeling of CaMnt1 in its apo form was performed using the AlphaFold2-mmseqs notebook provided by ColabFold [48]. The 1S4P structure was specifically provided as template and the resulting model was relaxed using the AMBER forcefield; all other parameters were kept in default.

It has been previously established that CaMnt1 uses preferentially methyl-α-mannose (MMA) or α1,2-mannobiose as acceptors when GDP-α-mannose (GDD) is given as donor sugar [32]. To achieve a model with GDD and MMA sugars bound to CaMnt1, three additional steps were performed. First, we used a local installation of AlphaFill to “transplant” the MMA, GDP, and MN cofactor from the 1S4P structure to the apo model [49]. This was only possible because we manually included MMA in the list of candidate ligands searched by AlphaFill (see the af-ligands.cif file). Next, we searched the PDB by filtering structures containing either GDD + MN, GDP-P’-mannopyranosyl + Mn^2+^ (GDX + MN) or UDP-alpha-mannose + Mn^2+^ (UFM + MN) as ligands. A total of four structures were retrieved, three of which bore GDD (7XJV, 5MM0 and 6YV9) and one that bore GDX (2BO8). These four structures were superimposed onto the AlphaFill model of CaMnt1 by means of their active site, using the MCSalign program to structurally superimpose the GDD and GDX ligands to the transplanted GDP. MCSalign superimposes two small-molecule selections based on Maximum-Common-Substructure. Manual adjustments were performed in PyMOL to superpose the Mn^2+^ ions while keeping the nucleosides as much superposed as possible. Finally, we “explanted” the GDP and MN from the AlphaFill model and created four models of CaMnt1 bearing GDD + MN or GDX + MN from each of the retrieved structures. All hydrogen atoms were removed, and the models were submitted to energy minimization using YASARA [50]. The model deriving from 7XJV displayed the lowest energy and highest Z-score, and therefore was selected as the final model. The overall quality analysis of the model was evaluated by the QMEAN Structure Assessment server (http://swissmodel.expasy.org/qmean/cgi.index.cgi) [51, 52]. The graphical representations of the modeled structures were generated using PyMOL (Schrödinger, http://www.pymol.org).

## Results

### Heterologous expression of Mnt1 from *Candida albicans* in *Komagataella phaffii*

The production of recombinant CaMnt1 was carried out through the heterologous expression in *K. phaffii*. Since mannosyltransferases are themselves glycoproteins [40], this expression system has great advantages, because it can perform post-translational modifications such as disulfide bridge formation, glycosylation, mannosylation, and folding of complex polypeptides [53]. *K. phaffii* is considered an easy microorganism to manipulate: it metabolizes methanol as the only source of carbon [54]; does not grow in a medium containing glucose [55]; and can express heterologous proteins under the control of the AOX promoter. In this expression system, proteins can either be expressed in an intracellular environment or secreted into the culture medium. The latter methodology greatly facilitates the purification of recombinant proteins [56].

**Fig. 1:**
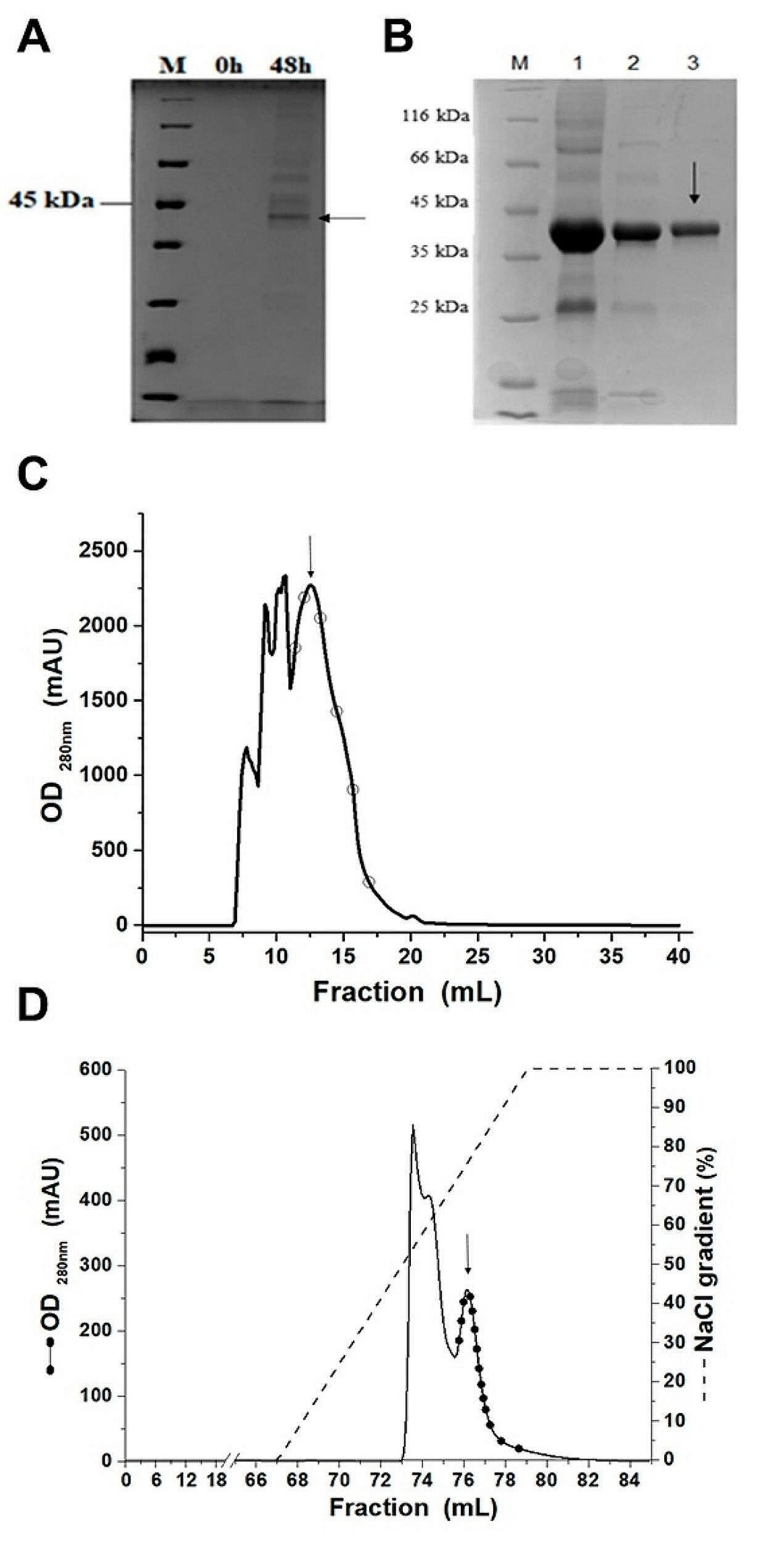
Expression and purification analysis of Mnt1 from *C. albicans.* **(A)** Induction time to 0 h and 48 h. **(B)** Purification profile in SDS PAGE 12%. Lane M: Molecular weight standard (Thermo Scientific). Lane 1: concentrated crude extract. Lane 2: S75 column CaMnt1 peak. Lane 3: Q XL column CaMnt1 CaMnt1 peak. **(C)** Chromatogram of the SEC S75 column where the empty circles represent the fractions collected. **(D)** Chromatogram of the IEX Q XL column, where the full circles represent fractions of the pure protein

According to the manufacturer’s instructions, gene expression using pPICZ family vectors should be induced when the initial inoculum achieves a OD_600nm_ between 1.0 and 1.3. Instead, we induced gene expression at OD_600nm_ of 5.0 to increase the yield. The auxotrophic transformant was cultured in BMMY medium and supplemented with methanol twice a day, being 0.5% in the morning and 1% in the evening. The crude extract was collected after 48 h, in which case the final OD_600nm_ reached a value of 40. This yielded a total protein concentration of 605 µg/mL in the culture supernatant. The expression of recombinant CaMnt1 can be seen in (Fig. 1A), wherein it was found secreted in the culture medium. Although the supernatant did not display significant impurities, it was still necessary to carry out chromatography steps to obtain CaMnt1 with suitable purity for structural analyses.

### Purification of recombinant protein

The recombinant CaMnt1 protein was purified through two steps of chromatography: first SEC (Fig. 1B and C), then IEX (Fig. 1B and D). In SEC, the protein was eluted between 12 and 17 mL. In IEX, elution occurred between 350 and 420 mM of NaCl. SDS-PAGE results show that a protein with high purity was obtained, which was later confirmed as CaMnt1 using mass spectrometry (Additional file 1). The yield of purified protein was 130 µg/mL, despite substantial loss of the recombinant protein during the purification process.

### MALDI-TOF/TOF MS analysis

The purified recombinant protein was analyzed by mass spectrometry to confirm its identity and approximate molecular mass. Trypsin cleavage of the recombinant protein allowed the identification and sequencing of 5 peptides that were compared to NCBI_nr_ database. The peptides AGGFFYER, FESGFFWR, IIYGHSESYR, NSDLYSLAESIR, and QEILNDYDYYWR resulted in positive matches (100% identity) to the CaMnt1 protein (NCBI accession number XP_721742.1) (Additional file 1, inset A). The sequence alignment between CaMnt1 and the five peptides is depicted in Additional file 1 (inset B).

### Circular dichroism (CD) assays

**Fig. 2:**
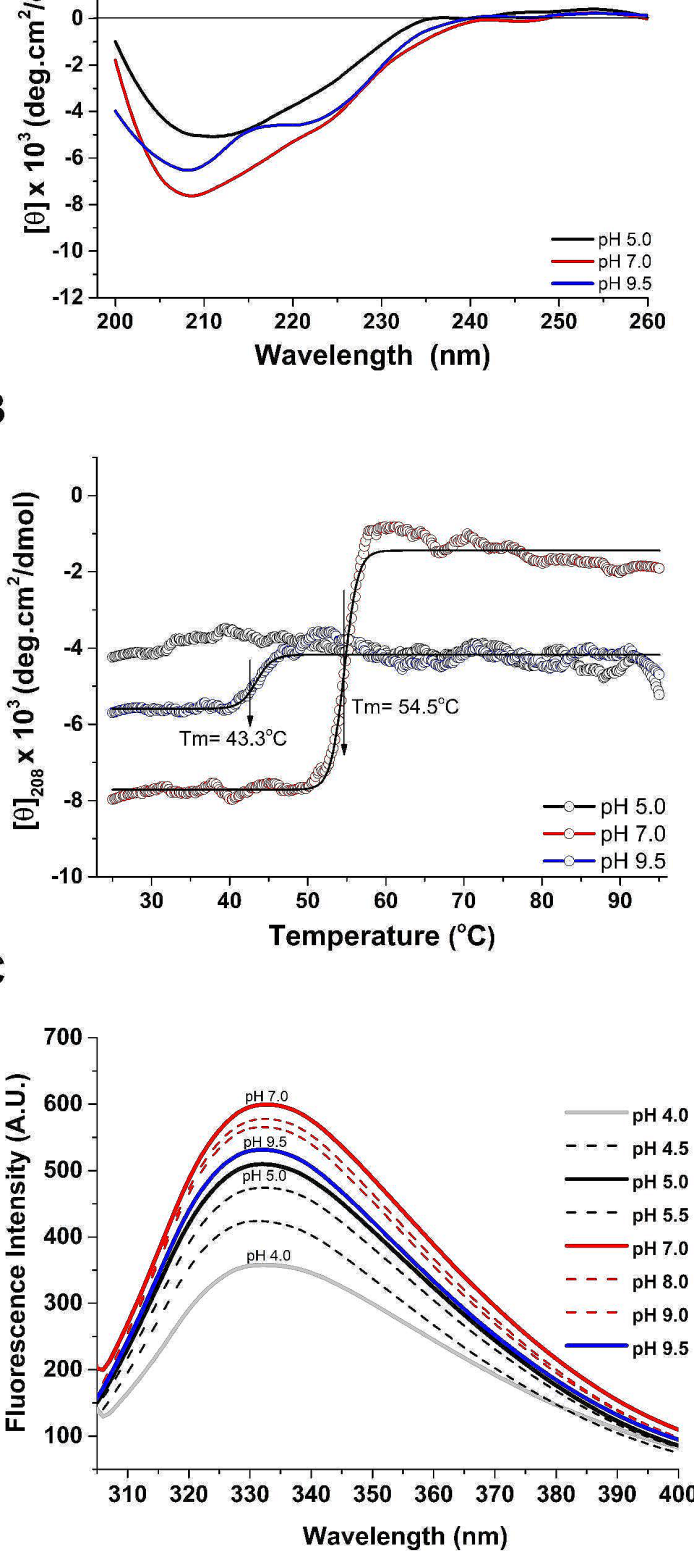
Structural characterization of CaMnt1 protein. **(A)** Far-UV CD spectra of CaMnt1 at pH 5.0, 7.0 and 9.5 at 25 °C. The CaMnt1 protein (0.11 mg/mL) was solubilized in 5 mM sodium citrate (pH 5.0), 5 mM Tris HCl (pH 7.0) and 5 mM glycine (pH 9.5). **(B)** Thermal denaturation profiles of MNT1. Unfolding curves of MNT1 (0.11 mg/mL) were monitored by changes of [θ]_208_ nm from 25 to 95 °C. The protein was solubilized in 5 mM sodium citrate pH 5.0 (black), 5 mM Tris HCl pH 7.0 (red line), and 5 mM glycine 9.5 (blue line). The arrows indicate a T_m_ of 54.5 °C at pH 7.0 and a T_m_ of 43.3 °C at pH 9.5. **(C)** Fluorescence emission of CaMnt1 as function of pH. Emission spectra changes were measured at different pH’s using 10 mM sodium citrate (pH 4.0‒5.5), 20 mM Tris HCl (pH 6.0‒9.0) and glycine (pH 9.5) with a range difference of 0.5. The emission bands centered at 332 nm displayed maximum fluorescence intensity at pH 7.0 (solid red line)

The Far-UV CD spectra of CaMnt1 exhibit different secondary structure profiles in acidic, neutral, and basic conditions at 25 °C, showing different intensities of the negative dichroic bands compatible with α-helix (208 nm and 222 nm) and β-sheet (218 nm) structures. At pH 7.0, more pronounced negative dichroic bands at 208 nm and 218 nm were observed than at pH 5.0 and pH 9.5, indicating a higher content of secondary structures at neutral pH (Fig. 2A).

The thermal unfolded curve and the Far-UV CD spectra of CaMnt1 showed small spectral changes at pH 5.0, indicating the prevalence of secondary structures (Fig. 2B and Additional file 2). However, the Far-UV CD spectra show a tendency of dichroic band shift from 208 nm to 218 nm (Additional file 2), compatible with an increase in β-sheets. At pH 7.0, the unfolding curve exhibited two states (native and denatured), with an abrupt change in the dichroic signal from − 8,000 deg.cm^2^/dmol to a value close to zero in the 45–65 °C range, and a T_*m*_ of 54.5 °C (Fig. 2B). Furthermore, the Far-UV CD spectra (as a function of temperature increase) show a strong change in the ellipticity signal at 208 nm, 218 nm and 222 nm, with values closer to zero (Additional file 2). These results indicate a complete unfolding of CaMnt1 over 60 °C at neutral pH. The unfolding curve at pH 9.5 shows a change of the dichroic signal from − 5,600 to approximately − 4,000 deg.cm^2^/dmol in the 25–55 °C range, indicating a partial denaturation state with a T_*m*_ of 43.3 °C (Fig. 2B). In this case, the Far-UV CD spectra show a tendency of dichroic band shift from 208 nm to 200 nm (Additional file 2), compatible with the increase of the unordered structure.

### Fluorescence spectroscopy

Conformational changes of CaMnt1 were analyzed by fluorescence measurements in acidic (pH 4.0–6.0), neutral (pH 6.5-7.0), and basic (pH 7.5–9.5) conditions. The fluorescence spectra show maximum emission bands centered at 332 nm, indicating that tryptophan (Trp) residues are buried within the hydrophobic environment of the protein in all the analyzed conditions (Fig. 2C). Although the emission bands were positioned at the same wavelength for all pHs, it was noted that the emission intensities peak at pH 7.0 and decrease in lower (4.0) or higher (9.5) pH, indicating a pH-dependent conformational change of CaMnt1.

### Molecular modeling of Mnt1 from *Candida albicans*

The structure of CaMnt1 (431 residues) was modeled using the AlphaFold2-mmseqs notebook provided by ColabFold. The donor (GDP-α-mannose; GDD) and acceptor (methyl-α-mannose; MMA) sugars, as well as the cofactor Mn^2+^ (MN), were added to the model using AlphaFill and MCSalign (see Methods). This model displayed 92% of the residues in energy-favored regions and 8% in permitted regions, which indicates good stereochemical quality. The first 65 residues participate in very few intramolecular interactions with the remainder of the protein, being composed of a large 35-residue-long loop and an N-terminal helix. This segment displayed the lowest confidence scores from AlphaFold2 and most likely represents an intrinsically disordered region (IDR). Therefore, we disregarded this region in our subsequent analyses.

**Fig. 3:**
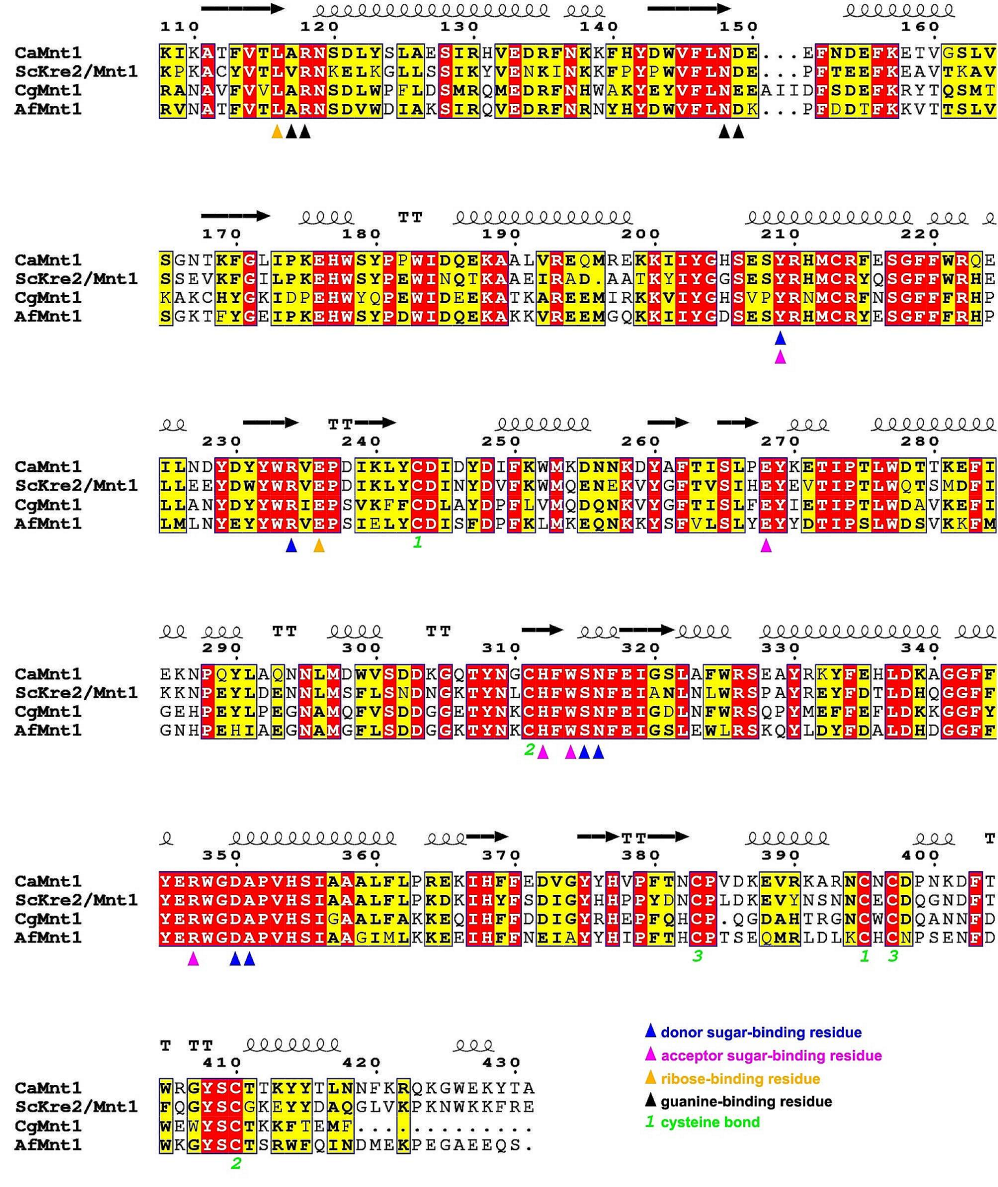
Multiple sequence alignment of Mnt1/Kre2 orthologs. *C. albicans* (UniProt accession Q00310); *S. cerevisiae* (UniProt accession P27809); *Cryptococcus gattii* (UniProt accession A0A0D0UYS7), formerly *Cryptococcus neoformans* var *gattii*; and *A. fumigatus* (UniProt accession Q4WV44). Triangles beneath sequences indicate substrate-binding residues: donor sugar-binding residues (in blue; same as in Fig. 4D), acceptor sugar-binding residues (in magenta; same as in Fig. 4A), ribose-binding residues (in yellow; same as in Fig. 4B), and guanine-binding residues (in black; same as in Fig. 4C). Cysteine bonds are indicated as green number pairs. The red areas indicate identical residues among all species, while yellow areas indicate similar residues. The alignment starts at residue 108 of CaMnt1 because this is the first residue of the Glycolipid 2-alpha-mannosyltransferase domain (Pfam 01793). Sequences were aligned using CLUSTAL W [57] and the final illustration was generated using ESPript [58]

Aside from the N-terminal IDR, the overall structure of CaMnt1 is nearly identical to the structure of Kre2/Mnt1 from *S. cerevisiae* (PDB 1S4P), which displays a GT-A fold (Additional file 3); GT-A proteins contain a single Rossmann fold domain while GT-B proteins contain two Rossmann fold domains. BLAST results indicate that both proteins share 60% identity (Fig. 3), thereby placing CaMnt1 in the GT-15 family of retaining glycosyltransferases.

**Fig. 4:**
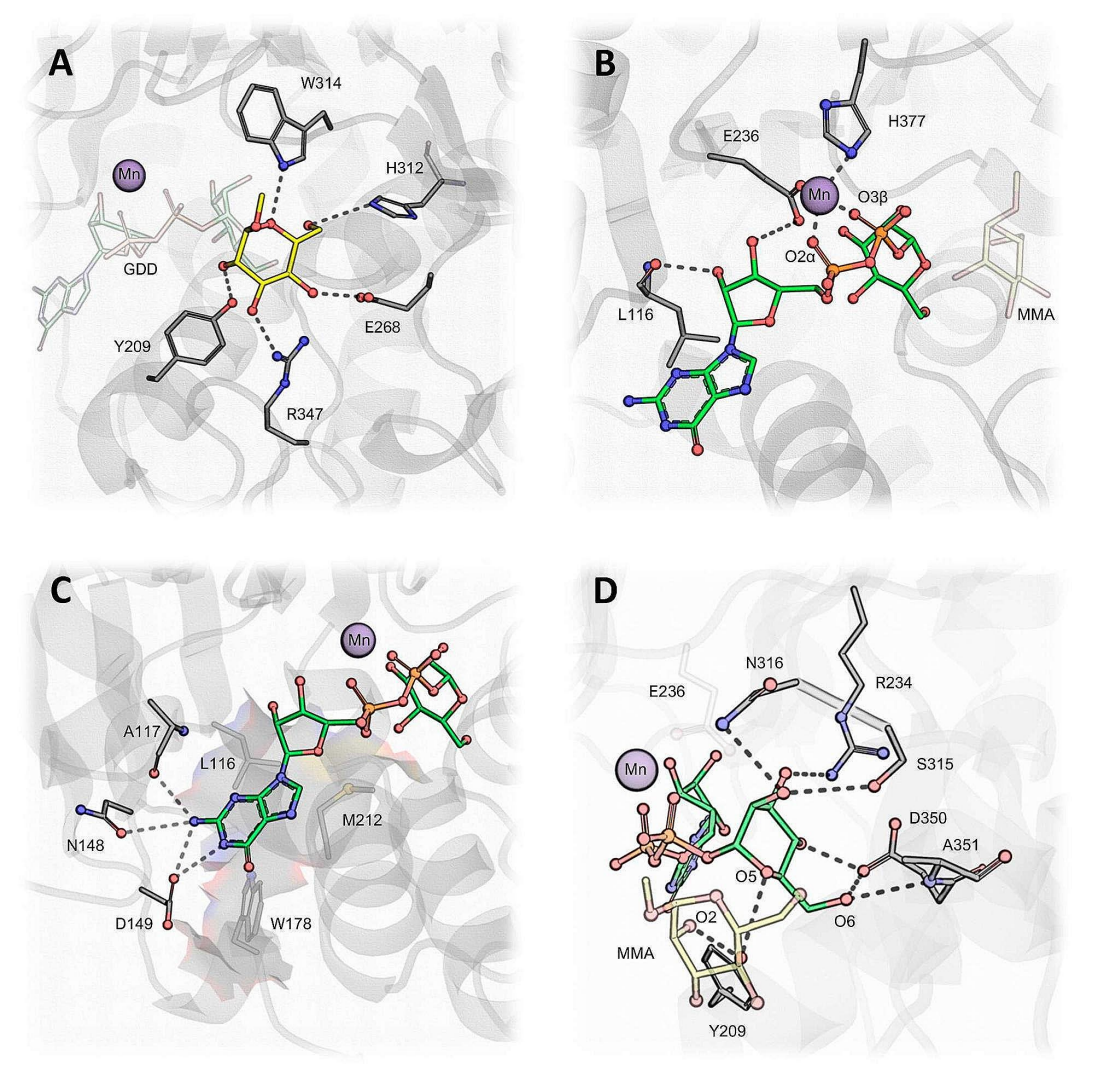
Ternary complex of CaMnt1 bound to the donor and acceptor sugars. **(A)** The acceptor sugar, methyl-α-mannose (MMA), is stabilized by five hydrogen bonds. His312, Trp314, Glu268, Arg347 and Tyr209 target the MMA O6, O5, O4, O3 and O2, respectively. The manganese ion (cofactor) is shown as a purple sphere, whereas the donor sugar, GDP-α-mannose (GDD), is shown in transparent ball-and-stick representation. **(B)** The ribose from GDD performs hydrogen bonds with Leu116 and Glu236, while the position of the manganese ion (transparent sphere) is coordinated by Glu236 and His377. **(C)** The guanine base is stabilized by hydrogen bonds with Ala117, Asn148, Asp149 and Arg 118 (not shown here for clarity’s sake; see Additional file 3). Leu116, Trp178 and Met212 create a hydrophobic pocket (in transparent surface representation) that accommodates the nucleoside moiety (nitrogenous base plus ribose) of GDD. **(D)** The donor sugar is coordinated by hydrogen bonds with Asn316, Arg234, Ser315, Asp350, Ala351 and Tyr209. The acceptor sugar (MMA) and Glu236 are shown in transparent ball-and-stick representation. The positions and charges of Glu236, Arg234 and Asp350 are conserved in retaining GTs, all of which use an equivalent of Asp350 to coordinate O6 of the donor sugar. Dashed lines indicate hydrogen bonds between donor and acceptor residues within 3.5 Å of each other

The metal- and acceptor sugar-binding pockets of ScKre2/Mnt1 and CaMnt1 are maintained by means of several conserved residues (Fig. 3), which include three disulfide bonds (C243-C395, C311–C410, and C383-C397). In particular, C311–C410 maintains His312 in contact with O6 from the acceptor sugar in the CaMnt1 structure (Fig. 4A). The remainder of the acceptor sugar is stabilized by Trp314, Glu268, Arg347 and Tyr209, which form hydrogen bonds with O5, O4, O3 and O2, respectively (Fig. 3A). Lastly, a cis-proline residue, Pro379, interacts with the methyl group (C7) in O1, and the aromatic ring from Tyr203 forms a weak interaction with O3 from the acceptor sugar by means of an O-H···π interaction (not shown) [59].

The metal-binding pocket of CaMnt1 lacks the canonical DXD motif found in other GTs [60, 61]. Instead, the manganese ion is kept in place by Glu236 and His377 (Fig. 4B). The phosphate oxygens O2α and O3β of Pα and Pβ further coordinate the Mn^2+^ ion.

We found that Arg118 interacts with GDD by means of its unique oxygen substituent at position 6 of guanine (Additional file 3). Additionally, Ala117, Arg118, Asn148 and Asp149 form specific hydrogen bonds with guanine in positions 1 (NH) and 2 (NH_2_), while Leu116, Trp178 and Met212 form a hydrophobic patch that accommodates the nucleoside moiety of GDD (Fig. 4C). In this case, the ribose portion of GDD is bound in the 3’-endo conformation, with the O2 and O3 forming hydrogen bonds with the main chain of Leu116 and the side chain of Glu236, respectively (Fig. 4B).

A particularly important residue for binding the donor sugar in CaMnt1 is Asp350, which coordinates both O4 and O6 (Fig. 4D). The donor mannose is further coordinated by hydrogen bonds with Tyr209, Arg234, Ala351, Ser315 and Asn316 (Fig. 4D).

## Discussion

We carried out the production of recombinant CaMnt1 in *K. phaffii* by inducing gene expression at an OD_600nm_ of 5.0 instead of the recommended values of 1.0 to 1.3 for pPICZ family vectors. This greatly increased the yield of recombinant protein in the culture supernatant after 48 h, in which case a total protein concentration of 605 µg/mL was obtained. This represents almost 10-fold higher production than those previously reported for this target [32, 35, 37]. For instance, Thomson [37] produced recombinant CaMnt1 in *K. phaffii* (strain GS115) using the p-HIL-S1 vector and obtained an optimum yield of 15–20 µg/mL in the culture supernatant after 5 days of methanol-induced expression (0.5% methanol every 24 h). Díaz-Jiménez [32] cloned CaMnt1 into the pPICZαC vector and transformed *K. phaffii* (strain X-33) to express the recombinant protein. Optimal induction for CaMnt1 was obtained after 48 h of incubation with 1% methanol, yielding 67 µg/mL in the culture supernatant. In both works, the analyses were performed using the crude extract rather than purified protein. Similarly, Lobsanov [35] obtained recombinant Kre2/Mnt1 of *S. cerevisiae*, a non-pathogenic fungus, using the pPICZαA vector and *K. phaffii* (strain KM71). The protein was purified by means of chromatography using Macro Prep ceramic hydroxyapatite and Q-Sepharose, but additional experimental details are missing. Finally, Wagener [38] carried out a cell viability assay for Mnt1 of *A. fumigatus*, but protein purification was unnecessary for the proposed objective. The results show that our protocol can substantially improve the yield of crude extract obtained from flask-grown cultures of *K. phaffi*, which is a limiting step for carrying out analyses that require a high degree of protein purity.

Recombinant CaMnt1 was purified through two steps of chromatography, which resulted in significant loss of the protein. Nevertheless, the yield of purified protein was still substantial, reaching an excess concentration of 130 µg/mL. Purification is a crucial step for the characterization of potential antifungal targets, which, once free of contaminants, can be employed in experiments such as circular dichroism, crystallography, and enzymatic activity assays. Importantly, this is the first purification protocol to yield sufficient quantities of CaMnt1 to allow elucidating key aspects of the protein’s structure and function. We confirmed the identity and approximate molecular mass of purified CaMnt1 using mass spectrometry. Therefore, the optimized expression and well-defined purification protocols established in this work pave the way to obtain pure CaMnt1.

According to the Far-UV CD spectra, CaMnt1 exhibits more pronounced secondary structures at pH 7.0 than at pH 5.0 and pH 9.5 (Fig. 2A). However, we observed complete unfolding of CaMnt1 over 60.0 °C at this pH (T_*m*_ of 54.5 °C; see Fig. 2B and Additional file 2). The prevalence of secondary structures below 45 °C and conformational changes observed above this temperature in neutral conditions (Fig. 2B and Additional file 2) concur with the optimal activity of CaMnt1 at 30 °C and pH 7.2 [32], which is far from its T_*m*_ of 54.5 °C. Interestingly, CaMnt1 sustains a partial denaturation state in pH 9.5 at temperatures over 45.0 °C (T_*m*_ of 43.3 °C; see Fig. 2B and Additional file 2), suggesting that it may transition into a molten globule state at basic pH.

The fluorescence spectra indicate that tryptophan (Trp) residues are buried within the hydrophobic environment of the protein in all the analyzed conditions (Fig. 2C). However, we observed a peak in the emission intensities at pH 7.0 with decreases towards acid (4.0) and basic (9.5) pHs, indicating a pH-dependent conformational change of CaMnt1. Generally, changes in emission intensity are correlated with conformational changes of Trp residues or residues around the Trp microenvironment [46]. Here, the variation of fluorescence emission intensities may have occurred due to conformational changes or the interaction between tryptophan residues and, most likely, the ionizable (pH susceptible) side chains of histidine and glutamic acid residues, or the N-terminus, all of which partially affect intramolecular interactions and directly affect the Trp microenvironment. These results corroborate those reported by Díaz-Jiménez [32] who demonstrated that CaMnt1 activity is pH dependent. According to fluorescence data, pH variation promoted conformational changes in the Trp microenvironment without modifying its position in the protein. Indeed, it is noteworthy that Trp233, Trp314 and Trp348 are closest to the active site’s Glu236, His312/Glu318, and Asp350, respectively (Fig. 4B). Therefore, these rearrangements of the Trp environment seem to be important for the protein to perform its biological function, since the optimum pH of 7.2 [32, 37] coincides with the highest fluorescence intensity compared to acidic and alkaline conditions (Fig. 2C).

The overall structure of CaMnt1 is nearly identical to the structure of Kre2/Mnt1 from *S. cerevisiae* (Additional file 3). BLAST results indicate that both proteins share 60% identity (Fig. 3), thereby placing CaMnt1 in the GT-15 family of retaining glycosyltransferases. However, sequence comparison between GTs can be misleading [60] and a structural assessment is necessary before classifying them as inverters or retainers. This classification infers that the transfer of mannose from the donor GDP-α-Man will result in a mannosylated product with the mannose attached by either an α-configured (retainer) or β-configured (inverter) glycosidic bond. The mannan structure in both *C. albicans* and *S cerevisiae* are formed mostly by α-(1–2) glycosidic bonds [29, 30] and both ScKre2/Mnt1 and CaMnt1 have been linked to α1,2-manosyltransferase activity [35, 36]. Furthermore, CaMnt1 has been shown to specifically use methyl-α-mannose and α1,2-mannobiose as acceptor sugars [37] and to preferentially add the second mannose residue during *O*-mannan synthesis [36], but possibly also the fourth and fifth mannose residues [32].

The metal-binding pocket of CaMnt1 lacks the canonical DXD motif found in other GTs [60, 61]. Instead, the manganese ion is kept in place by Glu236 and His377 (Fig. 4B). The phosphate oxygens O2α of Pα and O3β of Pβ further coordinate the Mn^2+^ ion. Contrary to the ternary complex of ScKre2/Mnt1 (PDB 1S4P), our model indicates that the phosphate groups of GDD do not form direct hydrogen bonds with the CaMnt1 structure. Instead, water molecules intermediate the interaction. This is partly due to the presence of the donor mannose, which is lacking in the 1S4P structure, and partly due to the substitution of Glu133 (in ScKre2/Mnt1) by Asp121 in CaMnt1. The bigger side chain of Glu133 is responsible for coordinating Arg130 towards the phosphate groups upon binding of the GDP moiety in ScKre2/Mnt1, which does not occur in CaMnt1 (Additional file 3). Rather, Arg118 in CaMnt1 (the equivalent of Arg130) can interact with the unique oxygen substituent at position 6 of guanine (Additional file 3). This suggests that CaMnt1 may display a higher preference for the guanine base than ScKre2/Mnt1.

The GDD’s ribose moiety was modeled in the 3’-endo conformation (Fig. 4B). This particular ribose conformation has been observed in intact donor substrates (PDBs 1ON6, 1GWV, and 1RF8), such as the one transplanted in CaMnt1, whereas the 2’-endo conformation has been observed in cleaved donor substrates (PDBs 1ON8, 1G93, and 1OQM), such as the one found in ScKre2/Mnt1. Both retaining GTs [62, 63] and inverting GTs [64] have been reported to readily use water as acceptor in enzyme-catalyzed hydrolysis of the donor substrate in the absence of a natural acceptor, although this feature is normally associated with a retaining reaction mechanism.

Interestingly, the representatives of all the retaining GT-A fold GTs (families GT-6, 8, 15, 27, and 64) display conserved three-dimensional features that differ from that of related inverting transferases. These features are also present in CaMnt1. In particular, the GTs from these families share an aspartate at the N-terminal of an alpha-helix (Asp350 in CaMnt1) which coordinates O4 and, most importantly, O6 of the donor sugar (Fig. 4D). Additionally, the same negatively charged residue that binds the metal ion (Glu236 in CaMnt1) also coordinates O3 of the nucleoside ribose (Fig. 4B), and a positively charged residue (Arg234 in CaMnt1), which creates a saline bridge between these two carboxylate residues, coordinates O3 and O4 of the donor sugar (Fig. 4D). Lastly, all known retaining GT-A fold GTs incorporate at least one histidine side chain into the divalent metal ion coordination (His377 in CaMnt1). Therefore, our observations suggest that CaMnt1 is a *bona fide* retaining GT-A fold mannosyltransferase.

Apart from the aforementioned Asp350 and Arg234 residues, the donor mannose is further coordinated by hydrogen bonds with Tyr209, Ala351, Ser315 and Asn316 (Fig. 4D). Tyr209 is particularly important because its equivalent in ScKre2/Mnt1 (Tyr220) has been implicated in the catalytic mechanism, with additional support being provided by mutagenesis data [35]. The mechanism of retaining GTs remains a subject of some debate and there have been several possible mechanisms suggested. The double-displacement mechanism involves the formation of a covalent glycosyl enzyme intermediate at the β-face of the C1” anomeric carbon and the release of the nucleoside diphosphate, followed by the subsequent attack of the glycosyl enzyme intermediate by the acceptor mannose at the α-face [65]. An alternative S_N_i-like mechanism involving an asynchronous front-face attack and the formation of an oxonium ion-like transition state has also been proposed for some glycosyltransferases [66–68]. Finally, the third possible catalytic mechanism is that of substrate-assisted catalysis, where an acceptor group other than O2 attacks the β-face of C1” anomeric carbon with subsequent attack at the α-face by the O2 of the acceptor mannose [66].

Our results show that the C1” anomeric carbon of the donor mannose is not within hydrogen-bonding distance of the Oη of Tyr209 to support its role in a double-displacement mechanism. Furthermore, the Oη of Tyr209 is nowhere near the β-face of the C1” anomeric carbon, from which the first of any putative double-displacement nucleophilic attack should occur. In any case, to serve as a nucleophile, the hydroxyl group of Tyr209 must be activated (deprotonated) by a catalytic base. No such residue is near Tyr209 to serve as a catalytic base. Instead, Tyr209 forms a hydrogen bond with O2 of the acceptor mannose at the α-face of the anomeric carbon (Fig. 4D). This interaction supports the role of Tyr209 in stabilizing the formation of an oxocarbenium ion-like intermediate while the acceptor sugar performs a nucleophilic attack at the α-face of the C1” anomeric carbon. As for the third mechanism, we note that O6 of the acceptor sugar could potentially perform an attack on the β-face of the anomeric carbon, thereby allowing O2 to attack at the α-face. However, in such a mechanism, the acceptor and donor sugars would be bonded, one way or the other, throughout the entire reaction. The fact that the donor mannose was never found bound to GTP or to the acceptor mannose in the ScKre2/Mnt1 structure (PDB 1S4P) suggests that the mechanism of action involves a concerted but dissociative and asynchronous nucleophilic attack. Therefore, while we are not able to rule out the third mechanism, we strongly favor the S_N_i-like hypothesis.

Notably, all but one of the substrate-binding residues described here are strictly conserved in *Candida* species. The exception is Asp149 (Fig. 4C), which is substituted by a glutamic acid residue in 9 out of 15 *Candida* species that we analyzed (Additional file 4). This substitution can be considered conservative since it maintains an acidic residue with a carboxylate group in the side chain. In addition, the substrate-binding residues are similarly conserved in *S. cerevisiae*, *C. gattii*, and *A. fumigatus* (Fig. 3), with only Asp149 (D/E) and Ala117 (A/V) showing some variability. Importantly, Ala117 interacts with guanine via its main chain (Fig. 4C), such that a mutation to valine is unlikely to affect the interaction with the substrate. Although *C. albicans* translates 97% of the CUG codons into serine instead of leucine [69], the gene that encodes CaMnt1 (GenBank accession no. X99619) does not have any CUG codon in its translated reading frame. Specifically, the leucine residue which we describe as important for substrate binding (Leu116) is encoded by the UUG codon. Therefore, Tyr209 and the other substrate-binding residues described here should provide a starting point for mutagenesis studies aiming to investigate the role of CaMnt1 in drug sequestration or biofilm formation.

## Conclusion

In this work, we have established an optimized protocol for the expression and purification of recombinant CaMnt1. To the best of our knowledge, this is the first description of purification steps using chromatography and the highest ever expression yield obtained for this protein. Our results show that recombinant CaMnt1 was obtained for the first time with a high degree of purity. Additionally, we have analyzed the structure of CaMnt1 using Far-UV CD and fluorescence spectroscopy experiments. The combination of these methods coupled with molecular modeling has allowed us to acquire important knowledge about the structure and function of CaMnt1. These data pave the way for the rational development of drugs targeting CaMnt1.

### Electronic supplementary material

Below is the link to the electronic supplementary material.

Additional file 1

Additional file 2

Additional file 3

Additional file 4

Additional file 5

## Data Availability

All data are included in the article and/or supplementary material. The producer clone and cloned pPICZαC vector are available upon request. The MNT1 gene from C. albicans is available under Genbank accession no. X99619. The structure model of CaMnt1 bound to its substrate and cofactor (ternary complex) is provided in the supplementary material as a PDB file (see Additional file 5).
