# Supplementary material for: An improved expression and purification protocol enables the structural characterization of Mnt1, an antifungal target from Candida albicans
Source: Fungal Biol Biotechnol. 2024 May 7;11:5. doi: 10.1186/s40694-024-00174-5 (PMC11077754; doi:10.1186/s40694-024-00174-5)
Supplement: Supplementary file 1 — Additional file 1 [file 40694_2024_174_MOESM1_ESM.pdf]

A

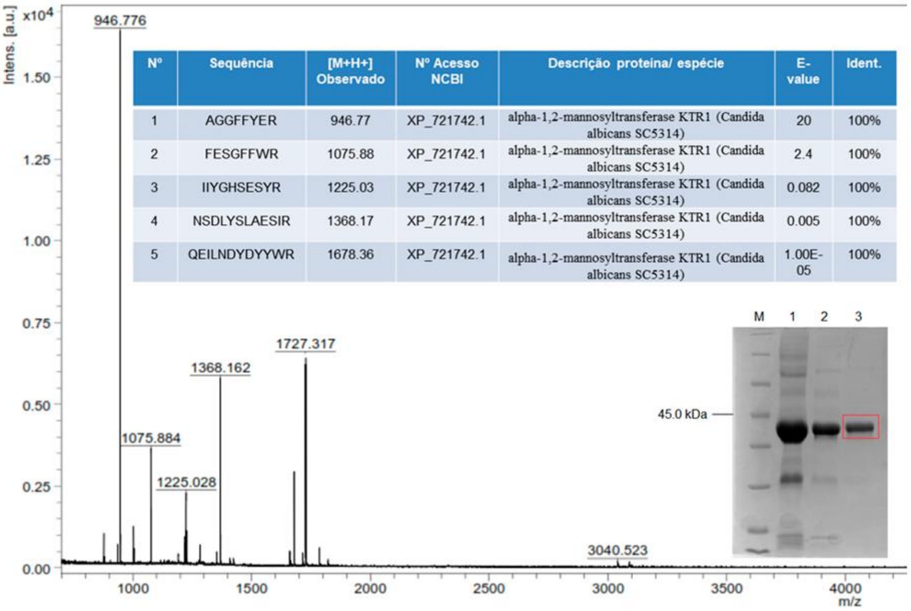

Nº	Sequência	[M+H+] Observado	Nº Acesso NCBI	Descrição proteina/ espécie	E-value	Ident.
1	AGGFFYER	946.77	XP_721742.1	alpha-1,2-mannosyltransferase KTR1 (Candida albicans SC5314)	20	100%
2	FESGFFWR	1075.88	XP_721742.1	alpha-1,2-mannosyltransferase KTR1 (Candida albicans SC5314)	2.4	100%
3	IIYGHSESYR	1225.03	XP_721742.1	alpha-1,2-mannosyltransferase KTR1 (Candida albicans SC5314)	0.082	100%
4	NSDLYSLAESIR	1368.17	XP_721742.1	alpha-1,2-mannosyltransferase KTR1 (Candida albicans SC5314)	0.005	100%
5	QEILNDYDYYWR	1678.36	XP_721742.1	alpha-1,2-mannosyltransferase KTR1 (Candida albicans SC5314)	1.00E-05	100%

B

```
CLUSTAL O(1.2.4) multiple sequence alignment


Pep5      ------------------------------------------------------------     0
Pep4      ----------------------------------------------------------NS     2
Pep3      ------------------------------------------------------------     0
Pep2      ------------------------------------------------------------     0
Pep1      ------------------------------------------------------------     0
Cal       GSGSAGAQVPLGKNRGPIPKAIMGAGEGGSDAPVPQQDIPDSYTLNDKIKATFVTLARNS   120

Pep5      ------------------------------------------------------------     0
Pep4      DLYSLAESIR--------------------------------------------------    12
Pep3      ------------------------------------------------------------     0
Pep2      ------------------------------------------------------------     0
Pep1      ------------------------------------------------------------     0
Cal       DLYSLAESIRHVEDRFNKKFHYDWVFLNDEEFNDEFKETVGSLVSGNTKFGLIPKEHWSY   180

Pep5      ------------------------------------------QEILNDYDYYWR------    12
Pep4      ------------------------------------------------------------    12
Pep3      --------------------IIYGHSESYR------------------------------    10
Pep2      ----------------------------------FESGFFWR------------------     8
Pep1      ------------------------------------------------------------     0
Cal       PPWIDQEKAALVREQMREKKIIYGHSESYRHMCRFESGFFWRQEILNDYDYYWRVEPDIK   240

Pep5      ------------------------------------------------------------    12
Pep4      ------------------------------------------------------------    12
Pep3      ------------------------------------------------------------    10
Pep2      ------------------------------------------------------------     8
Pep1      ------------------------------------------------------------     0
Cal       LYCDIDYDIFKWMKDNNKDYAFTISLPEYKETIPTLWDTTKEFIEKNPQYLAQNNLMDWV   300

Pep5      ------------------------------------------------------------    12
Pep4      ------------------------------------------------------------    12
Pep3      ------------------------------------------------------------    10
Pep2      ------------------------------------------------------------     8
Pep1      ---------------------------------------AGGFFYER-------------     8
Cal       SDDKGQTYNGCHFWSNFEIGSLAFWRSEAYRKYFEHLDKAGGFFYERWGDAPVHSIAAAL   360
```

Figure S1. **Mass spectrometry of the Mnt1 protein from *C. albicans***. (A) Representative graph of the identified peptides. At the top is the blast of each peptide sequence and the accession number. On the right is the SDS-PAGE gel 12% stained by Coomassie Blue R-250, with the sample sequenced. (B) Alignment of the five peptides identified by MALDI-TOF/TOF with CaMnt1 sequence. In red, the peptides and the corresponding region in the protein sequence.
