# Supplementary material for: An improved expression and purification protocol enables the structural characterization of Mnt1, an antifungal target from Candida albicans
Source: Fungal Biol Biotechnol. 2024 May 7;11:5. doi: 10.1186/s40694-024-00174-5 (PMC11077754; doi:10.1186/s40694-024-00174-5)
Supplement: Supplementary file 2 — Additional file 2 [file 40694_2024_174_MOESM2_ESM.pdf]

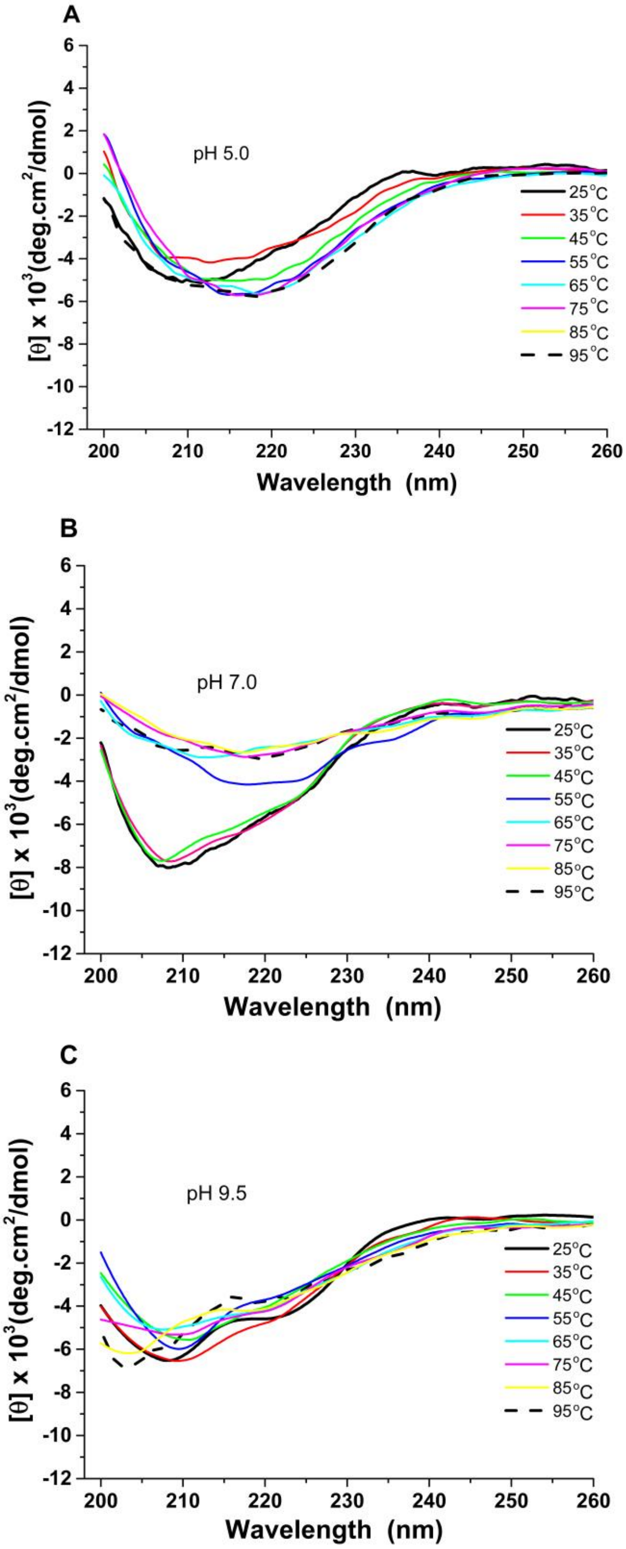

Figure S2. Far-UV Circular Dichroism (CD) spectra of CaMnt1 as a function of temperature. The Mnt1 protein (0.11 mg/mL) was solubilized in 5 mM of: sodium citrate pH 5.0 (**A**), Tris HCl pH 7.0 (**B**) and glycine pH 9.5 (**C**).
