# Supplementary material for: An improved expression and purification protocol enables the structural characterization of Mnt1, an antifungal target from Candida albicans
Source: Fungal Biol Biotechnol. 2024 May 7;11:5. doi: 10.1186/s40694-024-00174-5 (PMC11077754; doi:10.1186/s40694-024-00174-5)
Supplement: Supplementary file 3 — Additional file 3 [file 40694_2024_174_MOESM3_ESM.pdf]

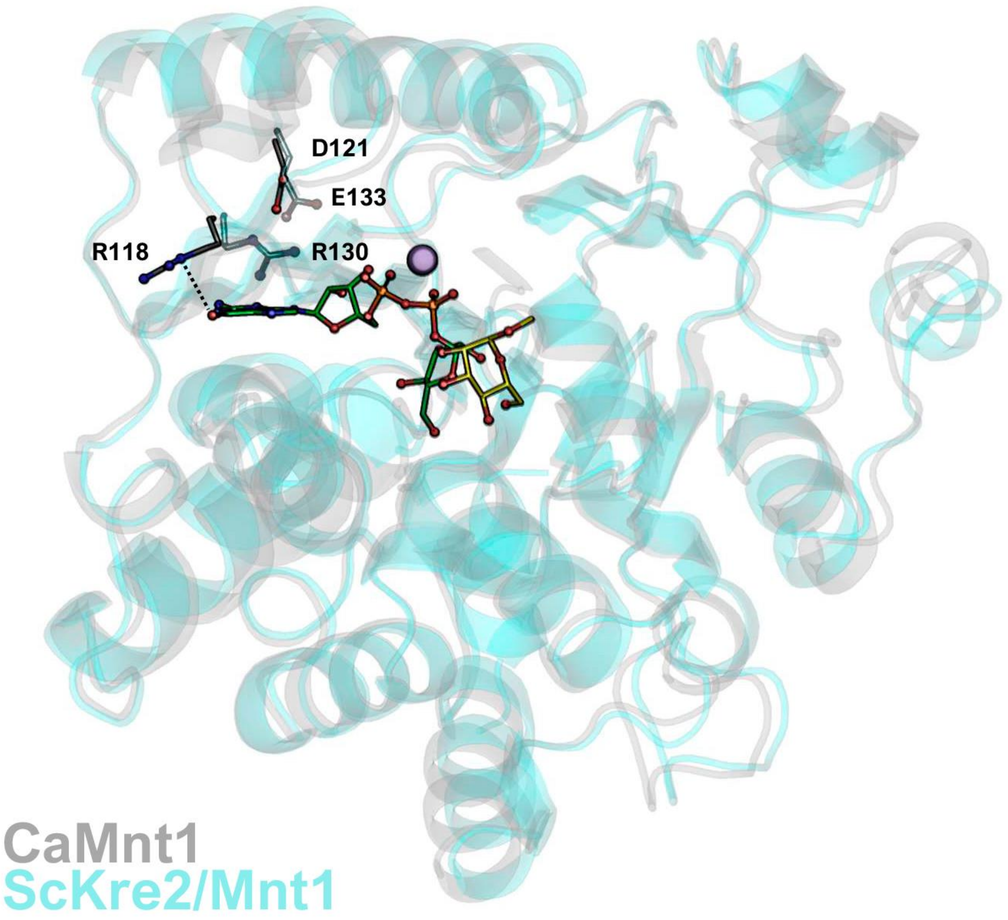

Figure S3. **Comparison between the ScKre2/Mnt1 and CaMnt1 structures**. The overall structure of CaMnt1 is nearly identical to Kre2/Mnt1 from *S. cerevisiae* (PDB 1S4P). However, due to the substitution of Glu133 (in ScKre2/Mnt1) to Asp121 (in CaMnt1), Arg118 from CaMnt1 makes specific contact with the oxygen substituent in position 6 of the guanine moiety. In contrast, Arg130 participates in a saline bridge with Glu133 in ScKre2/Mnt1.
