# Supplementary material for: An improved expression and purification protocol enables the structural characterization of Mnt1, an antifungal target from Candida albicans
Source: Fungal Biol Biotechnol. 2024 May 7;11:5. doi: 10.1186/s40694-024-00174-5 (PMC11077754; doi:10.1186/s40694-024-00174-5)
Supplement: Supplementary file 4 — Additional file 4 [file 40694_2024_174_MOESM4_ESM.pdf]

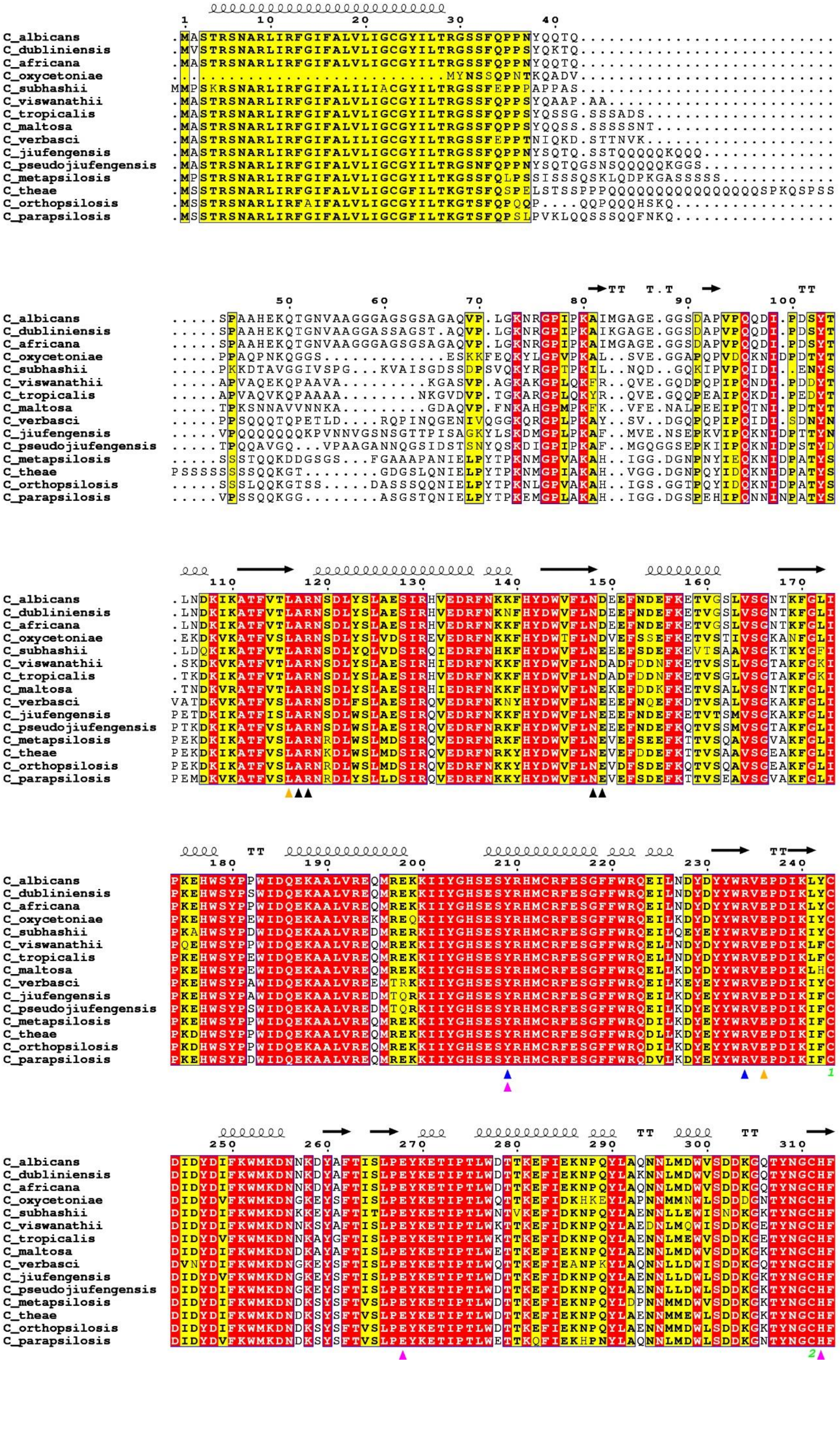
C_albicans
C_dubliniensis
C_africana
C_oxycetoniae
C_subhashii
C_viswanathii
C_tropicalis
C_maltosa
C_verbasci
C_jiufengensis
C_pseudojiufengensis
C_metapsilosis
C_theae
C_orthopsilosis
C_parapsilosis

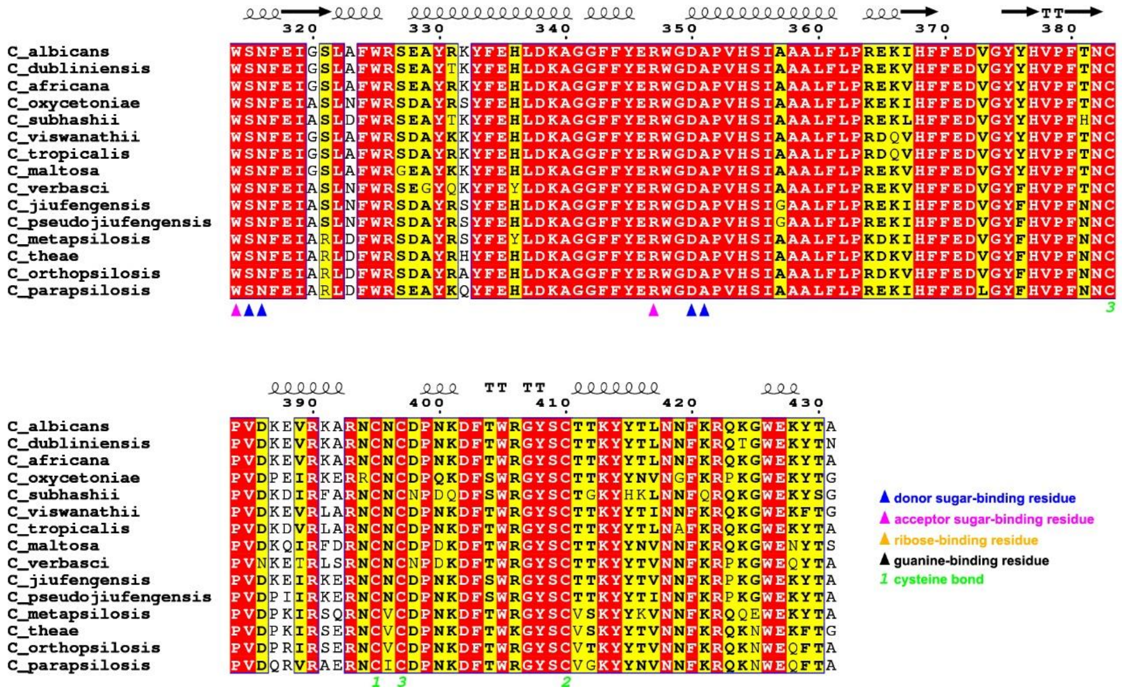

Figure S4. **Multiple sequence alignment of Mnt1/Kre2 orthologs in Candida species.** Triangles beneath sequences indicate substrate-binding residues: donor sugar-binding residues (in blue; same as in Fig. 4D), acceptor sugar-binding residues (in magenta; same as in Fig. 4A), ribose-binding residues (in yellow; same as in Fig. 4B), and guanine-binding residues (in black; same as in Fig. 4C). Cysteine bonds are indicated as green number pairs. The red areas indicate identical residues among all species, while yellow areas indicate similar residues. NCBI Protein accession numbers: *C. albicans* (XP_721742), *C. dubliniensis* (XP_002419113), *C. africana* (KAG8203359), *C. oxycetoniae* (XP_049178302), *C. subhashii* (XP_049260768), *C. viswanathii* (RCK67063), *C. tropicalis* (XP_002547836), *C. maltosa* (EMG49874), *C. verbasci* (CAI5758435), *C. jiufengensis* (XP_051619878), *C. pseudojiufengensis* (XP_051611761), *C. metapsilosis* (WOG36258), *C. theae* (XP_051610702), *C. orthopsilosis* (XP_003869631), and *C. parapsilosis* (KAI5908059). Sequences were aligned using CLUSTAL W [57] and the final illustration was generated using ESPript [58].
